# Experiences on health-related quality of life of Jordanian patients living with heart failure: A qualitative study

**DOI:** 10.1371/journal.pone.0298893

**Published:** 2024-04-18

**Authors:** Ahmad Rajeh Saifan, Haneen Abu Hayeah, Ateya Megahed Ibrahim, Alexandra Dimitri, Mahmoud Mohammad Alsaraireh, Hikmat Alakash, Nabeel Al Yateem, Donia Elsaid Zaghamir, Rami A. Elshatarat, Muhammad Arsyad Subu, Zyad Taher Saleh, Mohannad Eid AbuRuz

**Affiliations:** 1 Nursing College, Applied Science Private University Amman, Amman, Jordan; 2 Electronic Health Solutions Company, The University of Jordan, Amman, Jordan; 3 Nursing Department, College of Applied Medical Sciences, Prince Sattam Bin Abdulaziz University, Al-Kharj, Saudi Arabia; 4 Family and Community Health Nursing, Faculty of Nursing, Port Said University, Port Fuad, Egypt; 5 New York University Abu Dhabi, Abu Dhabi, UAE; 6 Princess Aisha Bint Al Hussein College for Nursing and Health Sciences, Alhussein Bin Talal University, Ma’an, Jordan; 7 Department of Nursing, University of Sharjah, Sharjah, United Arab Emirates; 8 Pediatric Nursing, Faculty of Nursing, Port Said University, Port Fuad, Egypt; 9 Department of Medical and Surgical Nursing, College of Nursing, Taibah University, Madinah, Saudi Arabia; 10 Faculty of Nursing and Midwifery, Universitas Binawan, Jakarta, Indonesia; 11 Department of Clinical Nursing, School of Nursing, The University of Jordan, Amman, Jordan; SPHMMC: St Paul’s Hospital Millennium Medical College, ETHIOPIA

## Abstract

**Background:**

Quantitative studies have provided valuable statistical insights into Health-Related Quality of Life (HRQoL) among patients with Heart Failure (HF), yet they often lack the depth to fully capture the nuanced, subjective experiences of living with HF particularly in the specific context of Jordan. This study explores the personal narratives of HF patients to understand the full impact of HF on their daily lives, revealing HRQoL aspects that quantitative metrics often miss. This is crucial in developing regions, where the increasing prevalence of HF intersects with local healthcare practices, cultural views, and patient expectations, providing key insights for tailored interventions and better patient care.

**Methods:**

Utilizing a phenomenological qualitative design, this study conducted face-to-face semi-structured interviews with 25 HF patients to deeply explore their lived experiences. Thematic analysis was employed to identify major themes related to their perceptions of HF as a disease, its impact on various HRQoL domains, and their recommended strategies to enhance HRQoL.

**Results:**

The study involved 25 participants (13 males, 12 females), aged 26–88 years (mean 63), with diverse education and heart failure (HF) severities. It revealed three themes: HF perceptions, its impact on health-related quality of life (HRQoL) across physical, psychosocial, spiritual, cognitive, and economic domains, and HRQoL improvement strategies. Participants had varied HF knowledge; some lacked basic understanding. The physical impact was most significant, affecting daily life and causing symptoms like breathing difficulties, coughing, edema, and fatigue. This physical aspect influenced their psychosocial and spiritual lives, cognitive functions, and economic stability, leading to fear, frustration, worry, social isolation, spiritual and cognitive challenges, and employment problems.

**Conclusions:**

The results underscores the need for holistic healthcare approaches, integrating medical, psychological, and social support. Key recommendations include integrated care models, comprehensive patient education, support networks, and policy interventions to enhance HF patient care.

## Introduction

In Jordan, noncommunicable diseases (NCDs) are a major health challenge, responsible for nearly 80% of all deaths, with cardiovascular diseases being a significant contributor, causing 42% of these fatalities [1]. A 2019 survey on NCD risk factors revealed high prevalence rates of hypertension (52%), diabetes (20%), and high cardiovascular disease risk (25%) among adults aged 45–69 years, indicating a pressing public health priority. Additionally, HF accounts for approximately 8.7% of deaths caused by all cardiovascular diseases in Jordan [2]. The prognosis of HF in the Middle East is concerning, with mortality rates of about 25% within one year and 50% within five years [3]. HF is a prevalent cardiovascular disease in Jordan, leading to a substantial number of hospital admissions and burdening the healthcare system due to its chronic nature and ongoing management requirements [4]. The condition has been associated with increased mortality rates and can result in life-threatening situations for affected individuals. The risk factors for HF in Jordan are likely similar to those observed in other countries and include hypertension, diabetes, coronary artery disease, obesity, and other cardiovascular diseases [5, 6]. The estimated prevalence of HF globally is 64.34 million cases, with contributing factors such as coronary artery disease, hypertension, valvular heart disease, and cardiomyopathies. Developing countries, including those in the Middle East, have seen a rise in HF diagnoses, with Saudi Arabia and Jordan reporting significant number of cases [4]. Understanding the epidemiology and prevalence of these contributing conditions is crucial for a comprehensive understanding of HF’s burden and management.

Traditionally, HF has been considered a disease primarily affecting the elderly population. However, recent trends indicate a shift, with HF increasingly impacting younger and middle-aged individuals. The average age of onset ranges from the late fifties to early sixties, indicating a significant change in the demographics of HF patients [7]. Interestingly, data from the latest HF registries in Middle Eastern Arab countries suggest that the average age of individuals affected by HF is at least 10 years younger compared to their Western counterparts [8]. These statistics underscore the growing prevalence and changing demographics of HF, necessitating a deeper understanding of the disease and its impact on individuals’ health and well-being. The specific context of Arab countries is of particular significance in understanding the prevalence of HF. The considerable number of reported cases and the increasing occurrence of HF among younger age groups highlight the urgent need for research and interventions to address this evolving public health challenge [9].

HF profoundly affects the health-related quality of life (HRQoL) of patients. HRQoL encompasses physical, psychological (emotional and cognitive), and social functioning and represents the patient’s overall perception of the impact of an illness and its treatment on their daily life [10]. Patients with HF experience lower HRQoL compared to individuals with other chronic diseases. The management of HF aims to improve both the physiological stability and HRQoL of patients. Moreover, HF patients often experience negative psychological manifestations, including depressive symptoms and poor overall quality of life. The impact on HRQoL is particularly pronounced in the physical domain among patients with moderate to severe depressive symptoms [11]. Considering the multi-dimensional and subjective nature of HRQoL, investigating patients’ perspectives is crucial for a comprehensive understanding of the disease’s impact on their lives [12]. While previous studies have objectively and statistically described HRQoL among HF patients using quantitative designs, some aspects remain poorly understood [13]. To fill these gaps, exploring HRQoL from patients’ perspectives is necessary, as it reveals how the disease truly affects their lives and helps identify interventions for improvement. It is essential to consider the cultural context, as HRQoL can be influenced by cultural factors. Most existing literature on HRQoL in HF has been conducted in Western contexts, necessitating research in specific cultural settings to capture the subjective experiences of patients.

Despite the significance of previous studies on HRQoL in HF patients, certain areas require further investigation. While these studies have shed light on HRQoL from an objective and statistical standpoint, the subjective experiences of patients regarding the impact of HF on their daily lives remain less explored. Furthermore, in Jordan, limited research has examined the HRQoL of patients diagnosed with HF and its relationship with socio-demographic factors and co-morbid diseases [14, 15]. However, clear culturally determined HRQoL cannot be transferred directly from Western culture to other cultural contexts. Therefore, a qualitative approach is crucial to explore the subjective experiences of HRQoL among patients diagnosed with HF in Jordan, allowing for the emergence of new themes from the data. By investigating the subjective experience of HRQoL among Jordanian patients diagnosed with HF, this study aims to provide a deeper insight into how the disease impacts their quality of life within the specific cultural context. The findings will contribute valuable insights into the unique challenges faced by HF patients in Jordan and inform the development of patient-centered interventions to improve their HRQoL.

### Study purpose and questions

This study aims to explore the personal experiences of HF patients in Jordan, focusing on their daily lived realities and perceptions of HRQoL. By employing a phenomenological approach, we seek to understand how these patients navigate their condition within their unique cultural context, encompassing emotional, cognitive, and social dimensions of HF. The main research question is: How do Jordanian patients diagnosed with HF perceive their HRQoL, particularly within their cultural setting? Additionally, we aim to investigate:

Patients’ perspectives on HF and its implications for their daily life.

The impact of HF on patients’ physical activity and psychosocial experiences.

Strategies identified by patients to enhance HRQoL in the cultural context of Jordan.

This culturally nuanced qualitative study is essential for uncovering the specific ways in which HF affects patients in Jordan, providing insights that are vital for patient-centered HF management and care strategies in this region.

## Methods

### Study design

This study utilized a qualitative research design employing a phenomenology research approach to address the research questions. Given the highly subjective nature of HRQoL, a qualitative approach was well-suited to provide a detailed and in-depth exploration of the individual’s subjective experiences [16]. This characteristic of the qualitative approach aligns with the study’s objective, which aims to understand the HRQoL from the perspective of patients diagnosed with HF. In the context this qualitative study, the phenomenology theory plays a pivotal role. This philosophical approach focuses on comprehending how individuals subjectively experience and interpret the world around them.

### Study participants

This study employed a purposive sampling strategy, targeting participants based on Jordanian nationality, age (18 years and above), and a confirmed diagnosis of HF (low or preserved ejection fraction (EF). This selection strategy also ensure including a diverse representation of our study cohort, we further ensured our sample included participants from various education level, socio-economic status, residential area (urban/rural), and with different durations of HF diagnosis (newly diagnosed versus long-term patients). This aimed to capture the varied experiences and impacts of HF across different social, economic, and geographical backgrounds, providing a more comprehensive understanding of the condition [17]. Additionally, participants needed to have a stable condition at the time of interviews and communicate in either Arabic or English. To avoid confounding the results, patients with comorbidities, such as cancer, psychiatric disorders, renal failure, or liver transplantation, were excluded. Excluding patients with comorbidities aligned with the study’s focus and ensured a clear understanding of HF’s direct impact on patients’ lives and the unique challenges they face in managing their condition and HRQoL. This decision was made to maintain the study’s clarity and validity in relation to HF in the context of Jordan. The inclusion of 25 HF patients was determined by data saturation, following Mason’s (2010) approach [18].

### Settings

The study was conducted in the outpatient cardiac clinics of four hospitals in Amman, Jordan, encompassing both public and private healthcare facilities. We selected two private hospitals and two governmental hospitals from a list of large hospitals in the capital city known for their comprehensive cardiac care services. The inclusion of both private and public hospitals was intentional to ensure a diverse representation of healthcare settings and patient demographics.

In terms of participant selection, we aimed for a balanced representation from each hospital. From the total pool of 25 participants, we evenly distributed the selection across the four hospitals. Approximately six participants were drawn from each private hospital and seven from each governmental hospital. This distribution was guided by the aim to capture a broad range of experiences reflective of different healthcare environments. Each of these cardiac outpatient clinics has a substantial patient load, with at least 50 patients attending daily, highlighting their significance as primary care points for HF patients. The diverse patient population attending these clinics provided a rich source for varied patient experiences, making them ideal settings for exploring the lived realities of individuals with HF in Jordan.

### Ethical considerations

This research was conducted with the approval of the Institutional Review Board (IRB) at the Applied Science Private University (Approval ID number: ASPU-2022-009) and the selected hospitals. Upon obtaining formal approvals, participants were approached at the clinic and presented with an invitation letter to join the study. They were also provided with an information sheet detailing the study’s objectives and procedures, after which they signed a written consent form. Prior to commencing the interviews, participants granted permission for tape-recording. Throughout the study, participants were assured of their autonomy to participate or withdraw their consent at any time without facing any penalty or harm.

To protect the privacy and confidentiality of participants, several measures were implemented. Participants were not required to provide their names, and all data records were stored in password-protected computers accessible only to the researchers. To maintain anonymity, interviewees’ real names were replaced with codes. Moreover, to ensure utmost privacy, all interviews were conducted in private spaces within the clinics. These measures were taken to ensure that participants’ personal information and experiences remained secure and confidential throughout the research process.

### Data collection

The selection of eligible participants was facilitated with the assistance of the head nurses of clinics, who reviewed the patient records and ensured that the participants met the predetermined eligibility criteria. Those who met the criteria and expressed their willingness to participate, providing informed consent, were interviewed in private settings within the selected clinics. The interviews were conducted by four authors of this study (AS, ZS, HA, MA), all of whom hold doctoral degrees in nursing and possess expertise in data collection and clinical qualitative research.

The data collection process took place from June 2022 to December 2022 and involved face-to-face semi-structured interviews. Data collection continued until data saturation was achieved, which occurred after the 24^th^ interview. An additional interview was conducted to confirm the decision to conclude data collection, as no new information emerged during the subsequent interview. These rigorous data collection methods ensured that a comprehensive and insightful exploration of the participants’ experiences was achieved, contributing to the robustness and validity of the study findings.

**Fig 1:**
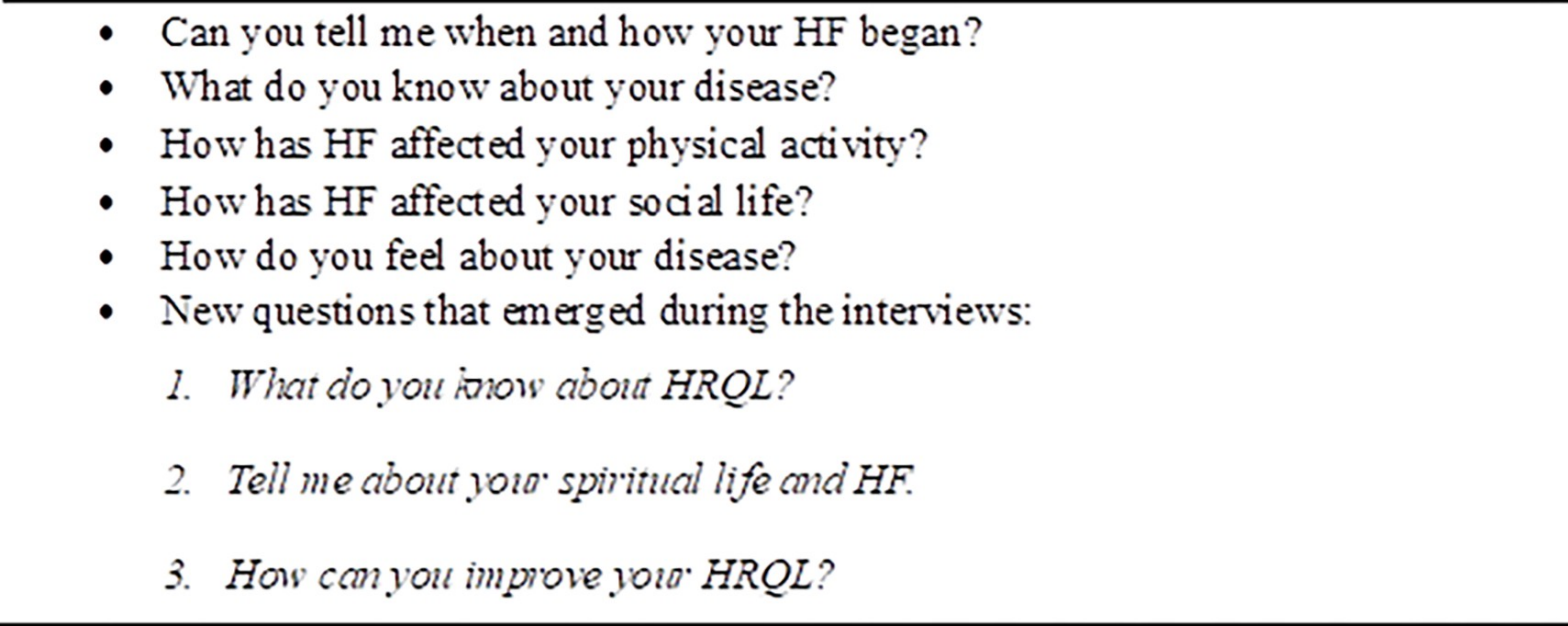
Initial and secondary interview questions.

During the interviews, which lasted between 40 to 60 minutes, audio recordings were made and later transcribed verbatim by the researchers. This meticulous approach allowed for a detailed analysis of the participants’ responses. The interview guide questions were carefully designed and are presented in Fig 1 to guide the interviews and ensure that relevant aspects were covered during the data collection process.

### Data analysis

We utilized Braun and Clarke’s (2006) thematic analysis method to examine our data, as detailed in Table 1 [19]. The transcription of interviews was done word-for-word by one author, while the rest participated in reviewing these transcriptions and annotating them. Our initial step in data analysis involved getting intimately familiar with the data, achieved by actively listening to the interviews and one author transcribing them. The next stage involved creating preliminary codes, with all transcripts being inputted into NVivo software, using version 8. During this, we meticulously read through all interviews line by line, constantly questioning, "What is the interviewee trying to convey with this statement?" We ensured every segment of the transcripts was coded.

**Table 1:** Phases of thematic analysis (Braun and Clarke, 2006).

No.	Phases of thematic analysis	Procedures and practical steps
**1.**	Familiarization yourself with your data	Transcribing data (if necessary), reading and re-reading the data, noting down initial ideas.
**2.**	Generating initial codes	Coding interesting features of the data in a systematic fashion across the entire data set, collating data relevant to each code
**3.**	Searching for themes	Collating codes into potential themes, gathering all data relevant to each potential theme
**4.**	Reviewing themes	Checking if the themes work in relation to the coded extracts (Level 1) and the entire data set (Level 2), generating a thematic ‘map’ of the analysis.
**5.**	Defining and naming themes	Ongoing analysis to refine the specifics of each theme, and the overall story the analysis tells, generating clear definitions and names for each theme.
**6.**	Producing the report	The final opportunity for analysis. Selection of vivid, compelling extract examples, final analysis of selected extracts, relating back of the analysis to the research question and literature, producing a scholarly report of the analysis

The subsequent phase focused on identifying themes, where codes were organized into a ’tree’ structure in NVivo to aid analysis, theme development, and theory formation. The fourth phase entailed revising these themes, refining the codes once all data was entered. The fifth stage was about defining and naming the themes, leading to the identification of five primary themes. These themes were then further integrated. The final stage in our analysis process was writing the report, beginning with drafting the results section.

All these steps, starting from initial data analysis to coding, were performed by one author (AS). In parallel, other authors (ZS, HA, MA) separately assessed the transcriptions, confirming the suitability of the analysis and themes. This led to minor modifications as we delved into the text’s subtleties. Table 1 details **phases of thematic analysis used in the analysis.**

### Rigor

To avoid their own bias in the data collection, analysis, and presentation of results, the research team took several steps. Firstly, they established the reliability of the gathered data through prolonged engagement with participants in the cardiology clinics, spending sufficient time with them to gain a deeper understanding of their experiences. Additionally, the team immersed themselves in the data by conducting thorough and repetitive readings of transcribed participants’ narratives, ensuring a comprehensive grasp of the content and context. To further enhance data credibility, the recorded data were repeatedly listened to and matched with the transcribed data and the emerging themes. This process aimed to confirm that the identified themes were firmly rooted in the participants’ experiences and not influenced by the researchers’ preconceptions. Moreover, the multiple authors involvement in the data collection and analysis ensured cross-checking findings, which minimized the potential for individual biases affecting the interpretation of the data.

By employing these rigorous measures, the research team sought to maintain objectivity and minimize the impact of personal bias at each stage of the study, from data collection to analysis and result presentation. These steps contributed to the overall validity and trustworthiness of the study’s findings.

## Results

### Study participants

In this study, we engaged 25 participants, including 13 males and 12 females, primarily married, with ages ranging from 26 to 88 years. Most had high school education or less, and they had been living with Heart Failure (HF) for durations between 2 to 12 years. The severity of their HF, indicated by ejection fraction, and the frequency of hospital admissions over the past year varied across the group.

Our qualitative analysis led to the identification of three major themes: Firstly, "Understanding and Reacting to HF: Varied Perceptions and Emotional Responses," which explored participants’ perceptions and emotional responses to their HF diagnosis. Secondly, "Interconnected Impacts of HF on Multiple Life Domains," highlighting how HF affected various aspects of their lives. Lastly, "Adaptive Strategies and Diverse Responses in Managing HF," focusing on the coping mechanisms and strategies used by participants to manage their condition.

These themes provide a snapshot of the multifaceted experiences of HF patients, capturing the complexity and diversity of their lives.

### Understanding and reacting to HF: Varied perceptions and emotional responses

Participants in this study exhibited a range of understandings and reactions to their Heart Failure (HF) diagnosis. Their perceptions varied from recognizing basic symptoms to grappling with a lack of comprehensive knowledge about HF. Many described HF in terms of weak heart muscles and fluid accumulation, as one participant stated:

"… All that I knowis that my heart muscles are weak. I am frequently admitted to hospital because of breathing difficulties… They used to tell me that there is fluid in my chest." (Participant (6), businessman)

However, there was a notable gap in detailed understanding, with some unaware of HF’s chronic nature, as evidenced by the surprise of one participant:

" What?! I did not know this. Are you serious that there is no cure for this disease?" (Participant 19, housewife)

Contrasting with the general lack of detailed information, a few participants actively sought out more in-depth knowledge about their condition and treatment options:

“… I asked my physician about my disease and its treatment. I read about HF, medications’ actions, and their side effects… I believe that the more I read about my disease, the more I can manage the symptoms…" (Participant (13), teacher)

Some interviewees viewed HF as an inexplicable disease, with one participant denying any predispositions despite a healthy lifestyle:

"… I used to walk more than six miles/day… I also worked in my farm… I did not know how my heart became weak… I had HF for five years." (Participant (7), farmer)

Emotional responses to HF diagnosis ranged from denial to acceptance and faithfulness. The shock of a young diagnosis and linking HF to pre-existing conditions were common themes:

"… Why did this happen to me? Why me…. I am still young… I am only 35……I was diagnosed with HF two years ago when I was only 33 years." (Participant (14), teacher)

This theme underscores the diversity in patients’ understanding of HF and their emotional journey post-diagnosis, revealing the complexities of living with a chronic heart condition.

#### Interconnected impacts of HF on Multiple Life Domains

This theme captures how HF deeply affects patients across physical, psychosocial, spiritual, cognitive, and economic dimensions. Participants shared vivid experiences of physical symptoms like difficulty breathing and fatigue, which not only hindered their physical activities but also led to broader psychosocial consequences:

"… I always feel that I cannot breathe; I need some air… my frequent hospital admissions are because of the fluid collection in my lungs… I barelycan get ready to pray or even to go to bathroom…" (Participant 23, housewife)

"… I feel my head is floating or as it is an empty skull… my hands and legs are cold… I feel like there is no blood there… my heart cannot work well…I sometimes cannot enjoy my days" (Participant (6), businessman)

The theme highlights the ripple effects of these physical challenges, leading to social isolation, emotional struggles, and diminished spiritual engagement. Participants described how their HF diagnosis disrupted their social lives and relationships, with some experiencing feelings of disappointment and frustration due to their inability to maintain previous lifestyle and spiritual practices:

"…I cannot pray while I am standing… I have to sit… I wish that God will accept my prayer as I am doing my best… They sometimes help me go to the bathroom or do ablution in bed… I used to go to the mosque for all prayer times. But now, I cannot do that which makes me feel disappointed…" (Participant 12, realtor)

Economic hardships and cognitive challenges, including difficulties with medication management and sleep disturbances, further exemplified the extensive impact of HF:

"… My health got worse; my heart became weaker… I am a driver. I cannot work anymore. I tried, but I could not do my job effectively. It needs a lot of effort. I am married, and I have two children. They are young; I am the only source of income. I sometimes receive financial aid." (Participant 4, driver)

"… I have difficulty falling asleep… I think this is because of overthinking; I think about everything and compare the past days with now, especially about my family life… after I got sick, l lost my job, and my son is working now, butwe cannot afford life expenses like before …." (Participant 15, housewife)

This theme illustrates the complex and interconnected nature of HF’s impact on patients’ lives, emphasizing the need for holistic management approaches that address these diverse challenges.

#### Adaptive strategies and diverse responses in managing HF

This theme encompasses the various strategies HF patients have adopted to improve their HRQoL, reflecting their personal experiences, advice from healthcare professionals, and other influences. Participants emphasized practical changes like short walks, healthy behaviors, and housing adjustments for convenience and stress reduction:

"I used to live in the third floor with no elevator. I could not go upstairs. I moved out to a first floor in another house. This was overwhelming." (Participant 3, driver)

Social and spiritual support also emerged as vital elements in enhancing HRQoL:

"Having someone caring about you is the most important thing that makes you control the challenges of the illness and tiredness… when I sit and talk with my family, I forget my tiredness." (Participant 20, housewife)

"… I go to church when I can, I feel peace and comfort praying and asking God for help and forgiveness… Faithfulness makes you stronger and overcomes any problem…" (Participant 8, teacher)

Job-related concerns were significant, with some participants advocating for employment considerations for those with HF:

"I work as a teacher. My work needs a lot of effort; I have difficulties managing it. I am still young, but I started thinking of quitting. I wish we had rules to consider our conditions so that we cankeep our jobs. This will make us feel better." (Participant 14, teacher)

In addition, the theme highlights nuanced differences in HF experiences influenced by age, gender, belief systems, economic status, and sociocultural factors. Younger participants reported more anxiety, while older individuals often accepted their diagnosis more calmly. The emotional impact of HF varied between male and female participants, and spiritual beliefs influenced coping strategies. Economic and sociocultural backgrounds also shaped access to healthcare and perceived quality of life:

“I’m just 40 and already struggling with HF. It’s like my whole future is uncertain now.” (Younger participant)

“At my age, health issues are expected. I’ve made peace with my HF.” (Older participant)

This theme illustrates the breadth of strategies employed by HF patients to manage their condition and the diverse ways in which individual factors shape their experiences and perceptions of living with HF.

## Discussion

This qualitative study provides insights into HF patients’ lived experiences in Jordan and their HRQoL. Participants described common HF symptoms, lacking adequate medical information, indicating the need for patient education and better communication with healthcare providers. The study emphasizes the importance of addressing physical, psychosocial, and economic aspects of HF to improve HRQoL, in line with previous research [20].

Health education regarding HF and its consequences, medication adherence, and lifestyle modifications can enhance HRQoL [21]. However, most participants in the study reported receiving relevant information about their condition from non-medical sources or through personal experiences rather than from healthcare providers. This lack of adequate information is consistent with previous studies in Jordan, where patients and family members often do not receive sufficient information about medical conditions due to a paternalistic approach by healthcare professionals. Effective communication with patients and their families is crucial in the caring process, and improving this aspect can positively impact HRQoL in HF patients [22].

Participants in the study highlighted the impact of HF on different domains of HRQoL. Most participants reported experiencing physical symptoms such as activity intolerance, dyspnea, fatigue, and edema, aligning with findings from previous studies that identified the physical domain as the most commonly affected aspect in HF patients [23]. Consequently, these physical symptoms logically contribute to the negative impact on HRQoL [24]. Moreover, the psychosocial domain was reported to be negatively impacted by HF, in line with prior research that found HF to have adverse effects on patients’ psychosocial lives [25]. The relationship between depression, anxiety, and HRQoL in HF patients is bidirectional, where depression and anxiety can influence physical activity and resultant HRQoL, while reduced HRQoL can lead to increased anxiety and depression [26]. These findings highlight the complex interplay between psychosocial well-being and overall HRQoL in the context of HF.

In the study, some participants contradicted the notion of HF’s negative impact on their social activities, attributing positive effects to the strong social support prevalent in Jordan [27]. A recent Ethiopian study also emphasized the significance of social support, as the absence of it was associated with poor HRQoL [28]. However, participants mentioned that HF had negative effects on their financial affairs due to increased medical expenses and job loss, aligning with previous research [15]. Jordan, a middle-income country with economic challenges, sees 20–33% of its population below the poverty line [29]. Factors like high public debt and regional instability have impacted the standard of living, influencing HRQoL for patients with chronic diseases like HF.

The study identifies the significant impact of HF on various domains of HRQoL, including physical, psychological, and social aspects. Physical symptoms such as difficulty breathing, edema, and fatigue were reported to have a considerable negative effect on participants’ daily lives. Spiritual activities were found to be coping mechanisms, positively influencing emotional well-being. Social support played a vital role in improving participants’ psychological and physical well-being and helping them adapt to their illness.

Cognitive impairment is a prevalent issue in HF, affecting memory and medication recall [30]. Sleep deprivation is also common in HF and linked to poor cognitive functions [31]. The study’s findings align with previous research, highlighting HF’s negative impact on cognitive aspects of HRQoL. Participants reported difficulties in medication intake recall and impaired concentration. Moreover, sleep disturbances were emphasized, further contributing to reduced HRQoL. Understanding and addressing cognitive and sleep-related challenges in HF patients are crucial for enhancing their overall well-being.

## Research implications and recommendations

The research focused on exploring the HRQoL of HF patients in Jordan using a qualitative phenomenological approach. Data were collected through in-depth interviews with HF patients to gain insights into their experiences, perceptions, challenges, and coping strategies related to the disease and its impact on various HRQoL domains.

The findings of this study have several implications for healthcare professionals, policymakers, and researchers. Firstly, it highlights the importance of health education for HF patients in Jordan. Improved patient education about the disease, its consequences, adherence to medications, and lifestyle modifications can enhance HRQoL. Healthcare providers should focus on effective communication with patients and their families to address information gaps and improve the overall caring process. Secondly, the research sheds light on the significant impact of physical symptoms on HF patients’ daily lives. Understanding the prevalence and severity of symptoms such as activity intolerance, dyspnea, fatigue, and edema can guide healthcare professionals in developing targeted interventions to alleviate these symptoms and improve HRQoL. Thirdly, the study emphasizes the psychosocial domain’s negative impact on HF patients’ HRQoL. The bidirectional relationship between depression, anxiety, and HRQoL highlights the need for comprehensive mental health support for HF patients. Integrating mental health services into HF management can help address psychological challenges and improve overall well-being.

Based on the research findings, several recommendations for future research can be made. Firstly, further quantitative studies should be conducted to quantify the prevalence and severity of physical and psychosocial symptoms in HF patients in Jordan. This can provide a more comprehensive understanding of the disease burden and identify specific areas for intervention. Secondly, longitudinal studies can be conducted to assess the long-term impact of HF on HRQoL and identify factors that predict positive or negative outcomes. Longitudinal data can help assess the effectiveness of interventions over time and guide personalized treatment plans for HF patients. Thirdly, comparative studies can be conducted to compare HRQoL among HF patients in Jordan with those in other countries or regions. This can help identify potential cultural or societal factors that influence HRQoL and inform cross-cultural interventions. Lastly, interventions that focus on social support and community-based care for HF patients should be explored. Understanding the role of social support networks and cultural norms in coping with HF can guide the development of community-driven interventions that enhance patients’ HRQoL and overall well-being.

## Study limitations

This study, while providing valuable insights into the experiences of HF patients in Jordan, has specific limitations that warrant consideration. One notable limitation is the exclusion of inpatients from our sample. This exclusion may have led to a lack of representation of the experiences of a significant subset of HF patients, potentially those with more severe conditions or different care needs compared to outpatients. Also, excluding patients with comorbidities, while aiding in isolating the impact of HF, also limits the scope of the study. Many HF patients have comorbid conditions, and their exclusion means the study may not fully represent the broader HF patient population, who often manage multiple health issues simultaneously. Also, conducting the study in Amman hospitals might have influenced the type of participants who were available and willing to participate. Patients attending these specific clinics might have different experiences compared to those receiving care in other settings especially the other less urban cities.

Furthermore, our study addressed the potential for researcher bias through the implementation of researcher triangulation, involving four different researchers in the interviewing and data analysis processes. This strategy likely reduced individual biases and enhanced the credibility of our findings by introducing multiple perspectives in interpreting the data. However, while this approach aids in mitigating bias, it may introduces another limitation concerning the consistency of data collection and interviewing. The presence of multiple data collectors, each with their personal differences, might have affected the interrater reliability. The use of standardized interview guide for interviewing hopefully has ensure more uniformity.

Lastly, our study focused solely on the experiences of HF patients. The inclusion of perspectives from healthcare providers and family members could provide a more holistic understanding of the care and support needs of HF patients, and should be considered in future research endeavors.

## Conclusion

Our study conducted in Jordan has yielded significant insights into the Health-Related Quality of Life (HRQoL) of Heart Failure (HF) patients. We found that physical symptoms such as difficulty breathing, coughing, edema, and fatigue severely impact patients’ HRQoL. These physical challenges often lead to psychosocial difficulties, including social isolation and emotional distress. Economically, HF places a burden on patients, affecting their ability to work and manage financial responsibilities. Additionally, cognitive impairment and sleep disturbances were identified as critical aspects of HRQoL affected by HF.

The implications of these findings for clinical practice are manifold. Healthcare professionals need to adopt a holistic approach to treating HF, which goes beyond managing physical symptoms to include support for the psychosocial, economic, and cognitive challenges faced by patients. This involves a multidisciplinary strategy incorporating patient education, symptom management, and mental health support.

Based on our findings, we recommend the following for enhancing the care of HF patients:

- **Integrated Care Models:** Develop care models that integrate medical, psychological, and social support to address the multifaceted challenges faced by HF patients.
- **Patient Education Programs:** Implement comprehensive patient education programs focusing on disease management, lifestyle modifications, and coping strategies for living with HF.
- **Support Networks:** Establish support networks involving healthcare providers, counselors, and peer groups to aid patients in navigating the emotional and social challenges of HF.
- **Policy Interventions:** Policymakers should consider these findings to develop policies that support HF patients, particularly in providing access to mental health services and financial assistance.

## Supporting information

S1 Checklist COREQ (COnsolidated criteria for REporting Qualitative research) checklist. (DOCX)
